# The recurrence or metastasis related gene predicts the prognosis of extremity and trunk soft tissue sarcoma

**DOI:** 10.1093/pcmedi/pbaf027

**Published:** 2025-10-28

**Authors:** Duo Wang, Dawei Sun, Jihao Tu, Xingyao Cui, Limei Qu, Lei Chen, Zhixin Zhang, Ziping Jiang, Ruijun Li, Zhaopeng Xuan, Jianli Cui, Xiguang Sun, Xiaoyan Jia, Pengcheng Liu, Ying Xiong, Jianing Wang, Yanfang Jiang, Bin Liu

**Affiliations:** Department of Hand and Foot Surgery, Orthopedics Center, The First Hospital of Jilin University, Changchun 130000, China; Engineering Laboratory of Tissue Engineering Biomaterials of Jilin Province, Changchun 130000, China; Department of Pathology, National University Hospital, 119074, Singapore; School of Disaster and Emergency Medicine, Faculty of Medicine, Tianjin University, Tianjin 300072, China; Institute of Disaster and Emergency Medicine, Faculty of Medicine, Tianjin University, Tianjin 300072, China; Medical School, Faculty of Medicine, Tianjin University, Tianjin 300072, China; Beijing ChosenMed Clinical Laboratory Co. Ltd., Beijing 100176, China; Department of Hand and Foot Surgery, Orthopedics Center, The First Hospital of Jilin University, Changchun 130000, China; Engineering Laboratory of Tissue Engineering Biomaterials of Jilin Province, Changchun 130000, China; Department of Hand and Foot Surgery, Orthopedics Center, The First Hospital of Jilin University, Changchun 130000, China; Engineering Laboratory of Tissue Engineering Biomaterials of Jilin Province, Changchun 130000, China; Department of Hand and Foot Surgery, Orthopedics Center, The First Hospital of Jilin University, Changchun 130000, China; Engineering Laboratory of Tissue Engineering Biomaterials of Jilin Province, Changchun 130000, China; Department of Hand and Foot Surgery, Orthopedics Center, The First Hospital of Jilin University, Changchun 130000, China; Engineering Laboratory of Tissue Engineering Biomaterials of Jilin Province, Changchun 130000, China; Department of Hand and Foot Surgery, Orthopedics Center, The First Hospital of Jilin University, Changchun 130000, China; Engineering Laboratory of Tissue Engineering Biomaterials of Jilin Province, Changchun 130000, China; Department of Hand and Foot Surgery, Orthopedics Center, The First Hospital of Jilin University, Changchun 130000, China; Engineering Laboratory of Tissue Engineering Biomaterials of Jilin Province, Changchun 130000, China; Department of Hand and Foot Surgery, Orthopedics Center, The First Hospital of Jilin University, Changchun 130000, China; Engineering Laboratory of Tissue Engineering Biomaterials of Jilin Province, Changchun 130000, China; Department of Hand and Foot Surgery, Orthopedics Center, The First Hospital of Jilin University, Changchun 130000, China; Engineering Laboratory of Tissue Engineering Biomaterials of Jilin Province, Changchun 130000, China; Department of Hand and Foot Surgery, Orthopedics Center, The First Hospital of Jilin University, Changchun 130000, China; Engineering Laboratory of Tissue Engineering Biomaterials of Jilin Province, Changchun 130000, China; Department of Hand and Foot Surgery, Orthopedics Center, The First Hospital of Jilin University, Changchun 130000, China; Engineering Laboratory of Tissue Engineering Biomaterials of Jilin Province, Changchun 130000, China; Department of Hand and Foot Surgery, Orthopedics Center, The First Hospital of Jilin University, Changchun 130000, China; Engineering Laboratory of Tissue Engineering Biomaterials of Jilin Province, Changchun 130000, China; Department of Hand and Foot Surgery, Orthopedics Center, The First Hospital of Jilin University, Changchun 130000, China; Engineering Laboratory of Tissue Engineering Biomaterials of Jilin Province, Changchun 130000, China; Department of Hand and Foot Surgery, Orthopedics Center, The First Hospital of Jilin University, Changchun 130000, China; Engineering Laboratory of Tissue Engineering Biomaterials of Jilin Province, Changchun 130000, China; Department of Hand and Foot Surgery, Orthopedics Center, The First Hospital of Jilin University, Changchun 130000, China; Engineering Laboratory of Tissue Engineering Biomaterials of Jilin Province, Changchun 130000, China; Department of Hand and Foot Surgery, Orthopedics Center, The First Hospital of Jilin University, Changchun 130000, China; Engineering Laboratory of Tissue Engineering Biomaterials of Jilin Province, Changchun 130000, China; Department of Hand and Foot Surgery, Orthopedics Center, The First Hospital of Jilin University, Changchun 130000, China; Engineering Laboratory of Tissue Engineering Biomaterials of Jilin Province, Changchun 130000, China

**Keywords:** soft tissue sarcoma, recurrence, multi-omics, prognostic model, relapse-associated risk score model, therapeutic targets

## Abstract

**Background:**

Relapsed soft tissue sarcomas (STS) have poor prognosis and limited treatment options. However, the molecular mechanism underlying recurrence and the prognostic predictor for STS are unclear.

**Methods:**

We enrolled 35 extremity and trunk STS patients. Tumor specimens of 20 relapsed and 15 primary STS underwent sequencing to detect DNA mutation, RNA expression, and DNA methylation. Moreover, 206 STS cases from The Cancer Genome Atlas (TCGA) were utilized to construct the relapse-associated risk score model (RRSM), validated using three Gene Expression Omnibus datasets. Key model genes, COL6A3, FZD7, ITPKA, and PRKAG1, were validated in formalin-fixed paraffin-embedded tissue sections from primary and relapsed STS patients, confirming their potential involvement in STS recurrence.

**Results:**

The primary STS exhibited an immune-enriched tumor microenvironment, whereas the tumor microenvironment of relapsed STS had features that promote tumor recurrence or metastasis. The RRSM could predict relapse-free survival in TCGA STS and performed well in the validation cohort. Multivariate analysis revealed that RRSM was an independent prognostic factor. Moreover, the nomogram developed had excellent predictive ability.

**Conclusions:**

This study revealed different multi-omic profiles between relapsed and primary STS. RRSM is a potential prognostic predictor for STS and lays a foundation for early intervention of high-risk STS patients. The expression of genes FZD7, ITPKA, and PRKAG1 may guide STS treatment decisions.

## Introduction

Soft-tissue sarcoma (STS) is a rare and heterogeneous malignant tumor originating from mesenchymal cells, constituting ∼1% of all adult cancers [1]. The extremities and trunk represent the most common primary sites of STS [2]. Surgical resection is the potentially curative treatment for primary non-metastatic extremity STS. However, local recurrence poses the main challenge for extremity STS patients who have undergone surgery resection. The risk factors for local recurrence include histological subtype, tumor size, anatomical location, and margin status [3, 4]. Most recurrences occur within the first 2 years after surgical resection, and local recurrence rates reached as high as 30% to 50% for extremity STS patients who underwent wide local excision [5, 6]. Despite various clinical approaches and limited systemic therapeutic options, patients with locally advanced or metastatic STS continue to have poor prognosis [7]. It is urgent to dissect the molecular mechanisms of relapse and develop personalized treatment for extremity and trunk STS patients.

On the genomic level, STS can be categorized into two molecular categories: those with complex karyotypes (aneuploidy and complex cytogenetic changes) and those with simple karyotypes (specific genetic variations, such as activating point mutation and translocations) [8]. Molecular genetic testing has emerged as a useful tool for the accurate diagnosis of sarcoma and appropriate clinical management [9]. Although the genomic and epigenomic profiles of pan-STS have been discussed in several large-scale studies, these findings have yet to be translated into routine clinical management [10–13]. The Cancer Genome Atlas (TCGA) consortium has delineated the integrated molecular landscape of 206 adult pan-STS representing six sarcoma subtypes and revealed that the immune microenvironment, inferred from DNA methylation or RNA profiles, was associated with prognosis [11]. In addition, high tumor mutational burden (TMB) was reported associated with worse disease-free survival (DFS) and remains significant after adjusting for risk groups based on Children’s Oncology Group risk stratification, fusion status, and chemotherapy regimens in rhabdomyosarcoma [14]. However, the potential molecular mechanisms of rapid postoperative relapse remain poorly understood. A thorough exploration of tumor recurrence could enhance our understanding of the mechanisms associated with tumor development and progression and aid in discovering more effective therapeutic strategies for STS.

In this study, we delineated the multi-omic profile of 35 extremity and trunk STS and compared molecular features between primary and relapsed STS. Here, we defined the cancer relapse-associated sub-pathway utilizing multi-omic data and developed a relapse-associated risk score model (RRSM) for STS prognosis by using the genes in the cancer relapse-associated sub-pathway. The RRSM offers insights for clinical risk stratification and identifies potential targets for precision therapy for STS patients.

## Materials and methods

### Samples and clinicopathologic data

In this study, we enrolled 35 patients with primary or relapsed STS of extremity and trunk who underwent surgical resection, and their diagnoses were confirmed through expert pathological review. The median age was 54 years, with a slight male predominance (62.9%). The majority (57.1%) had primary STS, while 42.9% had relapsed disease. Among the subtypes, myxofibrosarcoma was the most frequent, followed by dermatofibrosarcoma protuberans, myxoid liposarcoma, undifferentiated pleomorphic sarcoma, synovial sarcoma, and alveolar soft part sarcoma. Tumor locations were predominantly in the extremities (74.2%), with the remainder in the trunk (Supplementary Table 1, see online supplementary material).

### DNA-seq library preparation, sequencing, and variant calling

DNA was extracted from 35 fresh-frozen extremity STS samples, with genomic DNA from peripheral blood lymphocytes serving as the control. Genomic DNA was fragmented using an M220 Focused-ultrasonicator (Covaris) to generate 200–300 bp fragments. Fragmented DNA was ligated to indexed adapters using a KAPA Hyper Prep Kit (Kapa Biosystems). The DNA libraries were hybridized to ChosenMed panel (1123 genes) probes and RNA libraries were hybridized to ChosenMed RNA panel (89 genes) probes according to the manufacturer's instructions of the Fast Hybridization and Wash Kit (Twist Bioscience, Cat:101 175). The captured libraries were then sequenced on an MGISEQ2000 sequencing platform (MGI Tech Co., Ltd.) using paired-end 100 bp reads.

### Data preparation

The raw FASTQ files underwent quality control and cleaning using Fastp Software (Fastp, version 0.23.0). Then the clean reads were aligned to the human reference genome (UCSC hg19) using the Burrows–Wheeler Aligner (BWA version 0.7.11). The Genome Analysis Toolkit (GATK, version 4.2) module IndelRealigner and VarScan software were utilized to call somatic mutations, including small insertions, deletions, and single nucleotide polymorphisms (SNPs), both operated with their default parameters. All detected variants were annotated using ANNOVA filter, gene, and region based on other databases.

To refine the identification of single nucleotide variants (SNVs) and insertions and deletions, the following filtering criteria were applied to the mutation candidates: (i) population frequency < 0.001, (ii) variant allele frequency in the tumor sample > 0.05 and < 0.01 in the normal sample, and (iii) a total sequencing depth of at least 20 and at least 8 variant allele reads in the tumor sample, with a total sequencing depth of at least 20 and fewer than 25 variant allele reads in the normal sample.

The TMB is typically calculated by counting the number of non-synonymous somatic mutations found in the coding regions of genes sequenced with a given panel.

### Reduced representation bisulfite sequencing library preparation, sequencing, and data preparation

In the library preparation process of reduced representation bisulfite sequencing (RRBS), 1.5 μg of genomic DNA was digested using Mspl, with the addition of the appropriate amount of lambda DNA. The bisulfite conversion of DNA was treated with an EZ DNA Methylation-Gold^TM^ Kit (Zymo Research). DNA library quality was detected by Bioanalyzer 2100 (Agilent Technologies). RRBS was performed on an Illumina NovaSeq 6000, with paired-end reads of 150 bp length.

The sequencing reads were subjected to quality control using fastp (version 0.20.0.) [15] to filter out low-quality reads, trim adapter sequences, and remove other sequencing. The processed reads were aligned to the reference genome using Bismark [16]. Methylation calling was performed using the bismark_methylation_extractor tool, providing data on the methylation status at individual cytosine residues. Differential methylation gene analysis was conducted using the “DSS” package (version 4.4; 10.18129/B9.bioc.DSS). The DMLtest function was utilized to identify differential methylation gene loci between primary and relapsed STS.

### RNA-seq library preparation, sequencing, and data preparation

The mRNA tailed with poly-A was enriched by using Oligo(dT) from total RNA and then randomly fragmented, followed by reverse transcription to cDNA and double-strand synthesis. Subsequently, sequencing adapters were ligated and index PCR was conducted. Sequencing was performed on the Illumina NovaSeq 6000, with paired-end reads of 150 bp length.

Quality control of raw sequencing reads was performed using fastp (version 0.20.0.) [15]. The filtered reads were aligned to the human reference genome using HISAT2 [17]. Aligned reads were annotated and quantified using StringTie, with the Ensembl annotation file. Differentially expressed genes (DEGs) were identified out using the “DESeq2” package in R. Genes with an absolute log2 fold change ≥ 1 and *P*-value < 0.05 were considered significantly DEGs.

### Functional enrichment analysis

Gene Ontology, Kyoto Encyclopedia of Genes and Genomes [18], Gene Set Enrichment Analysis, as well as Hallmark analysis were performed using the “clusterProfiler” package [19, 20].

### Public data acquisition and preprocessing

RNA-seq data and the corresponding clinicopathological information of 206 STS samples were downloaded from TCGA (https://portal.gdc.cancer.gov/). Microarray expression profiling data and clinicopathological data of three STS cohorts (GSE21050, GSE71118, GSE71119) were obtained from the Gene Expression Omnibus (GEO) database. After excluding samples without survival information, these datasets retained 289, 312, and 123 samples, respectively. The “ComBat” tool was used to remove batch effects among these GEO datasets.

### Hub genes screening

Firstly, the Wilcoxon test was used to calculate *P*-values (p_RNA_) for the significance of differential expression between primary and relapsed STS for each gene in the training dataset. Similarly, *P*-values (p_meth_) were calculated for each gene in the RRBS data. Based on copy number variant (CNV) data, patients were divided into copy number variation and non-variation groups. The *P*-values (p_CNV_) of the DEGs between these two groups in the mRNA were subsequently calculated. After obtaining the *P*-value for each gene in the three omics analyses, we used “ICDS” R package (version 0.1.2) to integrate the *P*-values of the three omics and screen for differential sub-pathways between patients with primary and relapsed STS [21] and the association of genes and prognosis was further explored. The 206 TCGA STS samples with recurrence-free survival (RFS) information were used as a training dataset. The genes in the hub sub-pathway were subsequently analyzed via univariate Cox regression analysis. Genes with a *P* < 0.01 in the result were considered significant. Further screening was then performed using least absolute shrinkage and selection operator (LASSO) regression analysis, the results of which revealed candidate genes that correlated strongly with the prognosis of STS patients.

### Construction and validation of risk score signature

The risk score of each patient in the training dataset was calculated using the following equation.


\begin{eqnarray*}
{\mathrm{RRSM}} = \mathop \sum \limits_{\mathrm{i}} {\mathrm{Coef}} \times {\mathrm{Ex}}{{\mathrm{p}}^{{\mathrm{RNA}}}}
\end{eqnarray*}

Coef represents the coefficient of the candidate gene according to univariate Cox regression analysis of the RNA-seq data, and Exp^RNA^ represents the expression of the candidate gene in the RNA-seq data. Patients were divided into high-risk and low-risk subgroups based on the cut-off calculated using the “survminer” package (version 0.4.9; 10.32614/CRAN.package.survminer). Kaplan–Meier (KM) survival curves were plotted using the “survival” (version 3.3.1;10.32614/CRAN.package.survival) and “survminer” (version 0.4.9; 10.32614/CRAN.package.survminer) packages to demonstrate the difference in RFS between the high- and low-risk groups of the training dataset. The receiver operating characteristic curve (ROC) was then plotted using the “survivalROC” package (version 1.0.3) [22], and the area under the ROC curve (AUC) was considered to be an accurate predictor of patient prognosis for RRSM. The distribution of RRSM and RFS for both high- and low-risk groups of patients in the training dataset was then plotted, as well as a heat map of the expression levels of the central genes that make up the RRSM in the training dataset.

To validate the predictive ability of the RRSM beyond the training dataset, patients in the three GEO validation sets GSE71118, GSE71119, and GSE21050 were also categorized into high- and low-risk groups of patients using the RRSM, respectively, after which KM survival curves as well as ROC curves were plotted. Next, the “maftools” R package [23] was utilized to plot the mutations in the high- and low-risk groups of the training dataset and to compare mutated genes that were significantly different between the two groups.

### Immune microenvironment characteristics

In order to investigate the variations in the immune microenvironment among patients following classification based on RS (Recurrence Score) signature, we employed the CIBERSORT algorithm and the LM22 immune cell reference gene expression matrix from the TCGA training dataset.

### Survival model nomogram

To compare prognostic characteristics with other clinical indicators, we used the “regplot” package (version 1.1; https://rdrr.io/cran/regplot/) for gender, age, FNCLCC grade, tumor size, mitotic rate, margin of surgery resection, and RRSM to create nomograms for these clinical indicators to estimate DFS at 1, 3, and 5 years, and the “rmda” package (version 1.6; https://cran.r-project.org/web/packages/rmda/index.html) was used to create calibration and decision curve analysis plots and ROC curves to assess the validity of the nomogram.

### Drug sensitivity analysis

To obtain the differences in sensitivity to drugs between patients grouped by the RRSM, we obtained data from the Genomics of Drug Sensitivity in Cancer database, calculated the half-maximal inhibitory concentration (IC50) using the “oncoPredict” (version 0.2) R package, and used the t-test to obtain drugs that differed significantly between patients in the high- and low-risk groups.

### Immunohistochemistry

This study included 100 STS samples, comprising 50 primary and 50 recurrent cases. Tissue sections were obtained from the Department of Pathology at The First Hospital of Jilin University and its Lequn Branch. All cases were pathologically confirmed.

Immunohistochemistry was performed using primary antibodies against FZD7, ITPKA, PRKAG1, and COL6A3 (Servicebio, Wuhan, China). For each section, three random high-power fields (200×) were photographed and evaluated independently by two certified pathologists with associate senior titles. Both were blinded to the patients' clinical and transcriptomic data to ensure unbiased assessment. Image analysis was carried out using Image-Pro Plus 6.0. A consistent threshold for brown staining was applied across all images. The integrated optical density and area were measured, and the mean density (integrated optical density/area) was calculated to quantify protein expression.

### Statistical analysis

The survival curve was compared via the KM method and the log-rank test. When comparing continuous numerical values between two groups, we employed two statistical methods: the Wilcoxon test and the Student’s t-test. Specifically, when assessing the significance of the results, we set a *P*-value < 0.05 as the criterion to ensure that the differences obtained are statistically significant. All statistical analysis was conducted in R software (v.4.1.2).

## Results

### Study population and patient clinicopathological characteristics

This study recruited 35 patients with extremity and trunk STS, who underwent surgical resection for primary or relapsed disease at The First Hospital of Jilin University between 2022 and 2023 (supplementary Table 1). The median age of these participants was 54 (ranging from 28 to 92) years, with 62.9% (22/35) being male. Relapsed STS patients accounted for 57.1% (20/35), while the rest were diagnosed as primary STS. Myxofibrosarcoma and dermatofibrosarcoma protuberans were the predominant STS subtypes, comprising 28.6% (10/35) and 20% (7/35) of cases, respectively. The tumor location predominately occurred in an extremity (74.3%, 26/35), with the trunk accounting for 23.7% (9/35).

### Genetic features in relapsed and primary extremity and trunk STS

**Figure 1.**
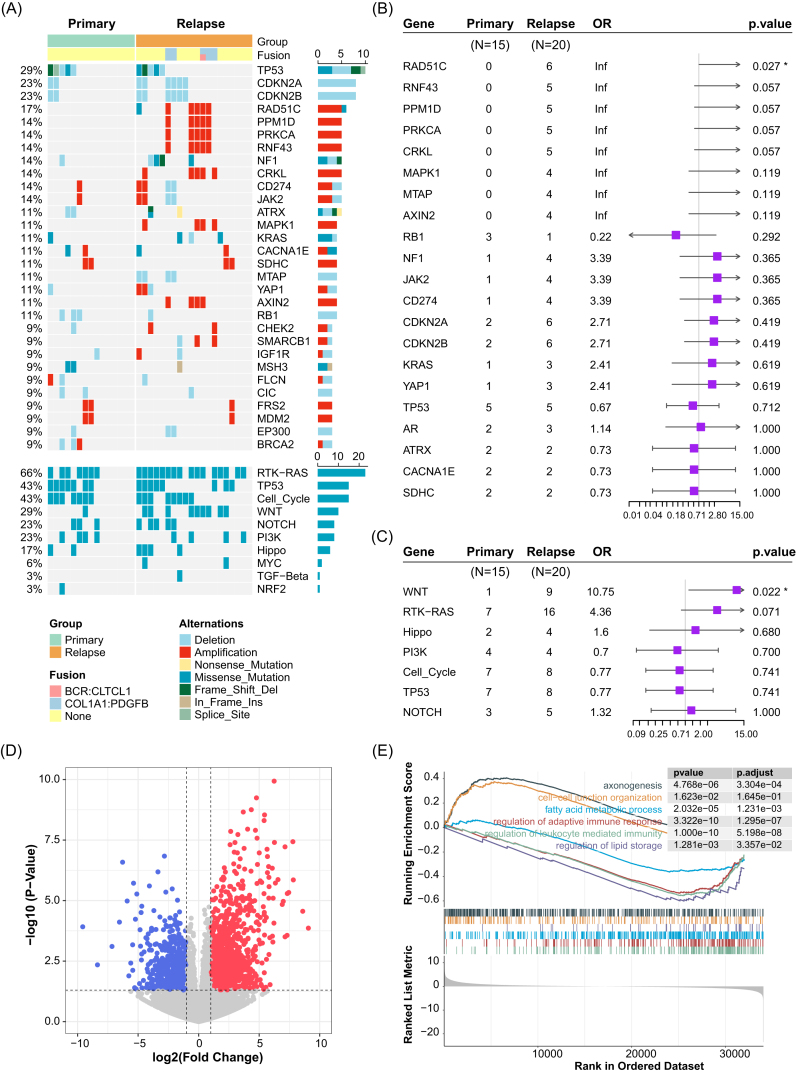
Landscape of 35 extremity and trunk STS patients between primary and relapsed groups (**A**). Forest plot of altered genes that occurred in primary and relapsed STS patients (**B**). Forest plot of ten signaling pathway alterations detected in primary and relapsed STS patients (**C**). Volcano plot of the DEGs between primary and relapsed STS patients (**D**). GSEA plot of hallmark pathways in comparisons between primary and relapsed STS patients (**E**).

We explored the difference in genomic profiles between relapsed and primary STS. The incidence and distribution of gene mutation and CNV are shown in Fig. 1A. The commonly mutated genes detected in the 35 STS cases were TP53 (6/35, 17.1%), NF1 (3/35, 8.6%), KRAS (3/35, 8.6%), and MSH3 (3/35, 8.6%). Genes with CNV that frequently occurred in >10% of cases were CDKN2A, CDKN2B, RAD51C, PPM1D, PRKCA, RNF43, CRKL, MAPK1, SDHC, MTAP, AXIN2, and RB1. In addition, five relapsed patients exhibited COL1A1-PDGFB fusion (supplementary Fig. 1A, see online supplementary material). Only RAD51C gene alterations were significantly more frequent in the relapsed group than in the primary group (Fig. 1B). In addition, the analysis of 10 signaling pathways revealed that the Wnt pathway had a markedly higher mutation frequency in the relapsed group (Fig. 1C). To further contrast genomic alteration profiles between fusion-positive and fusion-negative patients, we integrated targeted-panel sequencing data from 91 diagnostic outpatient STS samples (supplementary Fig. 1B). Fusion-positive tumours are characterised by frequent amplifications of MDM2, CDK4, EP300, and FRS2, whereas fusion-negative cases are dominated by mutations in TP53, NOTCH1, and related genes.

It is well known that STS generally has a low TMB. However, we noted that relapsed STS patients exhibited a higher, albeit not significantly different, TMB compared to the primary group (supplementary Fig. 1C). We then deciphered the mutation signatures of 35 STS patients, yielding four distinct signatures (supplementary Fig. 1D). These signatures exhibited high cosine similarity with established COSMIC mutational signatures including SBS1 (spontaneous deamination of 5-methylcytosine, clock-like signature), SBS5 (clock-like signature), SBS87 (thiopurine chemotherapy treatment), and SBS2 (activity of the APOBEC family of cytidine deaminases), respectively [24]. The relapsed STS tended to exhibit a more pronounced clock-like signature, SBS1 and SBS5, though this was not statistically significant (supplementary Fig. 1E).

### DEGs enriched in immune-related pathways in primary STS tumors

To elucidate the transcriptomic difference between relapsed and primary STS, we conducted GSEA functional and pathway enrichment analysis. There are 145 up-regulated DEGs and 216 down-regulated DEGs in relapsed STS (Fig. 1D). GSEA analysis of the DEGs suggested that primary STS were enriched in fatty acid metabolic process, regulation of adaptive immune response, regulation of leukocyte mediated immunity, and regulation of lipid storage pathways, while the relapsed group was associated with axonogenesis and cell–cell junction organization pathways (Fig. 1E). The gene ontology term enrichment analysis revealed that the up-regulated DEGs in relapsed STS were significantly enriched in extracellular structure organization, extracellular matrix organization, cell junction assembly, collagen-containing extracellular matrix, and growth factor binding pathways (supplementary Fig. 2A, see online supplementary material), while the up-regulated DEGs in primary STS, were associated with leukocyte migration, chemokine-mediated signaling pathway, cytokine receptor binding, phospholipase A2 binding, phospholipase activity, and lipase activity pathway (supplementary Fig. 2B). These results suggested that primary STS were prone to express the genes related to immune response, while relapsed STS overexpressed genes associated with extracellular matrix construction which could promote tumor recurrence and metastasis. Analysis of the cancer hallmark pathway revealed that epithelial–mesenchymal transition (EMT), hypoxia, and notch signaling pathways were significantly more activated in the relapsed group (supplementary Fig. 2C), while the primary group had more pronounced induction of immune-related pathways, including complement, inflammatory response, TNFA signaling via NFKB, and fatty acid metabolism pathway (supplementary Fig. 2D).

In addition, we identified the difference of the immune cells in the tumor immune environment (TME) between primary and relapsed STS. The infiltration of activated B cells, activated CD8 T cells, activated dendritic cells, effector memory CD4 T cells, gamma delta T cells, immature B cells, macrophages, myeloid-derived suppressor cells, natural killer T cells, neutrophils, type 17 T helper cells, and type 2 T helper cells were significantly higher in primary STS than in relapsed STS (supplementary Fig. 2E). This result indicated that primary STS was prone to be an “immune hot” tumor, while relapsed STS tended to show a relatively “immune cold” state.

### Methylation features in relapsed and primary extremity and trunk STS

A previous study demonstrated that the CpG methylation signature was associated with disease recurrence in early-stage hepatocellular carcinoma [25]. To explore whether similar methylation patterns were associated with the recurrence of STS, we compared the methylation profiles between two groups. In total, 6468 differentially methylated regions were found in 642 hypermethylated genes and 653 hypomethylated genes (supplementary Table 2, see online supplementary material). Gene ontology enrichment analysis showed that the hypermethylation genes in relapsed STS were enriched in the pathways including DNA-binding transcription depressor activity (supplementary Fig. 3A, see online supplementary material), whereas hypomethylation genes were associated with focal adhesion, transforming growth factor beta binding, cadherin binding, and actin binding pathways (Supplementary Fig. 3B).

### Construction of a recurrence-related prognosis model

**Figure 2.**
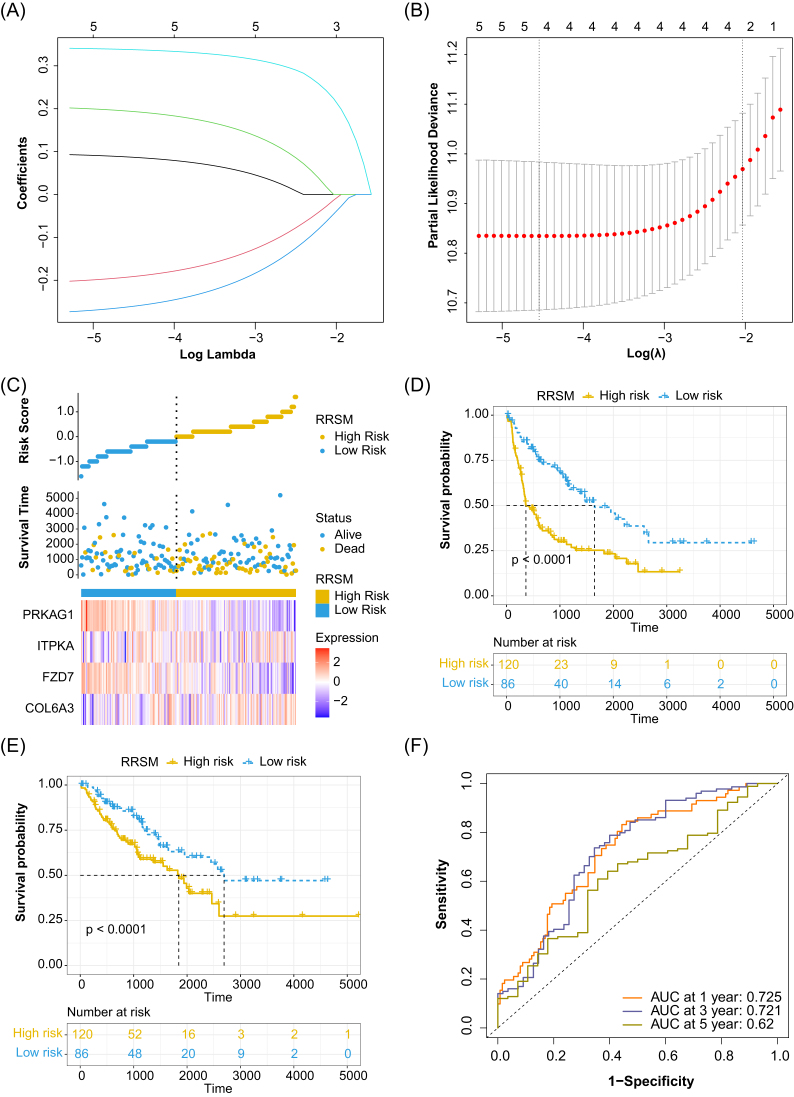
Partial likelihood deviance coefficient profiles (**A**). LASSO Cox analysis of the DEGs associated with the prognosis (**B**). Distribution of risk scores among the TCGA STS patients, patient survival status, and the heatmap of four genes in the TCGA training cohort (**C**). The *x*-axis is patients ID ranked by risk score from low to high. The *y*-axis is risk score, survival time (days), and four candidate genes. ROC curves for 1-, 3-, and 5-year RFS of TCGA STS patients (**D**). KM curve analysis for the RFS of high- and low-risk groups in TCGA STS patients (**E**). KM curve analysis for the OS of the high- and low-risk groups in TCGA STS patients (**F**).

We integrated multi-omics *P*-value (p_RNA_, p_meth_, and p_cnv_) (supplementary Table 3, see online supplementary material) and analyzed the sub-pathway by using the “ICDS” package. We examined the impact of sub-pathway genes (supplementary Table 4, see online supplementary material) on the prognosis of TCGA STS patients. Univariate Cox regression analysis was conducted to identify potential prognostic genes and the results suggested that 14 genes were significantly correlated with the RFS of TCGA STS patients (supplementary Table 5, see online supplementary material). Then we performed LASSO Cox regression analysis and selected five genes (*P* < 0.01) out of the 14 prognosis-related genes in the TCGA STS cohort (Fig. 2A and B). Finally, a four-gene combined model, containing *COL6A3, FZD7, ITPKA*, and *PRKAG1*, was termed the RRSM (RRSM = 0.21426*EXP^COL6A3^_–_ 0.26790*EXP^FZD7^ + 0.23888*EXP^ITPKA^_–_0.29810*EXP^PRKAG1^). The optimal cutoff value of the RRSM classified TCGA STS patients into high-risk and low-risk groups, between which the expression of the four genes were significantly different (Fig. 2C). Consistently, the high expression of *COL6A3* (supplementary Fig. 4A, see online supplementary material) and *ITPKA* (supplementary Fig. 4B) was associated with poorer RFS, whereas the expression of *FZD7* (supplementary Fig. 4C) and *PRKAG1* (supplementary Fig. 4D) was linked to better RFS. The high-risk score patients had a significantly poorer RFS (Fig. 2D) and overall survival (OS) (Fig. 2E) than the low-risk score group (*P* < 0.0001). In addition, the RRSM performed well for predicting the 1-, 3-, and 5-year RFS in the TCGA STS cohort (the AUC values for 1-, 3-, and 5-year RFS were 0.725, 0.721, and 0.620, respectively) (Fig. 2F).

### Performance of the RRSM in the validation cohort

**Figure 3.**
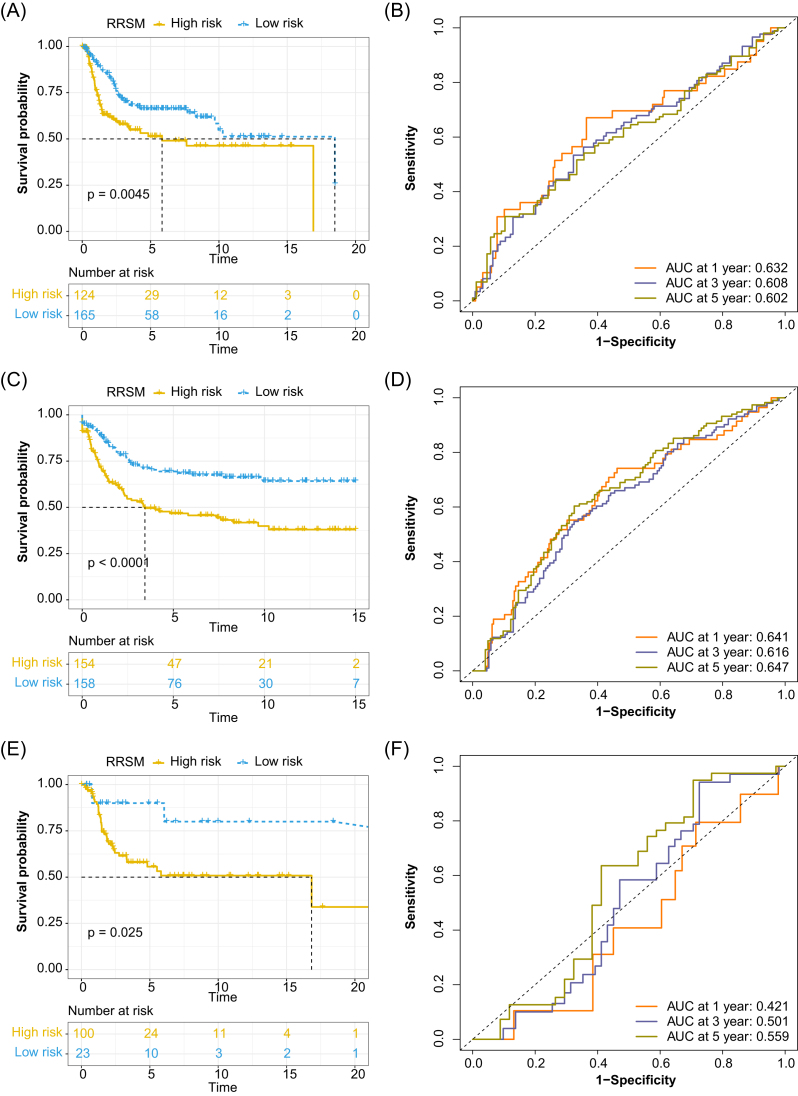
Validation of the prognosis prediction of RRSM in three GEO STS cohorts, including GSE21050, GSE71118, and GSE71119. KM survival analysis for the RFS of high- and low-risk groups in GSE21050 (**A**), GSE71118 (**C**), and GSE71119 (**E**). ROC curves for 1-, 3-, and 5-year RFS of GSE21050 (**B**), GSE71118 (**D**), and GSE71119 (**F**).

We validated the prognostic value of RRSM in three validation cohorts including GSE21050, GSE71118, and GSE71119. The patients in the three cohorts were classified into high-risk and low-risk using their optimal cutoff value for each validation cohort. Patients with high-risk score showed a markedly worse RFS than those in the low-risk score group (Fig. 3A, C, and E). The results also revealed that RRSM could effectively predict the prognosis in the validation cohorts, with the risk score effectively forecasting 1-, 3-, and 5-year RFS (Fig. 3B, D, and F).

### Molecular and immune features of different risk score groups

**Figure 4.**
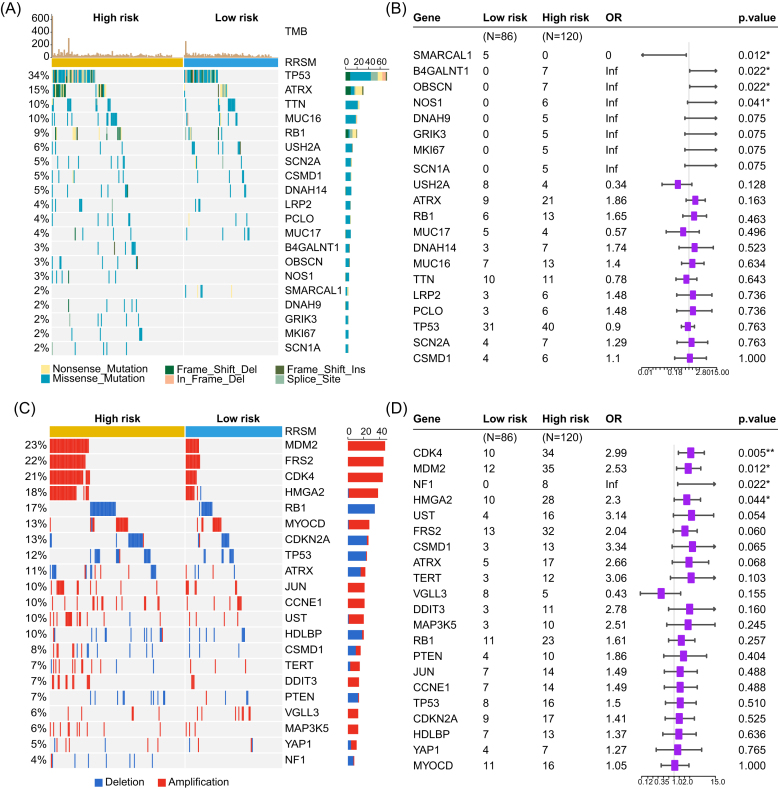
Mutation landscape in TCGA-STS, showing the top 20 frequently mutated genes in RRSM high- and low-risk groups. The top bar chart illustrates the total mutation counts per sample. The rightmost bar chart represents the number of each mutation type in each gene (**A**). Genes with significantly different mutation rates between RRSM groups (**B**). The top 20 CNV of the RRSM groups (**C**) and significantly different CNV distribution (**D**).

We investigated the relationship between RRSM and other molecular features (Fig. 4A) and found that a higher proportion of *B4GALNT1, OBSCN* (Obscurin, cytoskeletal calmodulin and titin-interacting RhoGEF), and *NOS1* (Nitric Oxide Synthase 1) mutations were identified in the high-risk score group than in the low-risk score group, while patients with a low-risk score had more *SMARCAL1* mutation than those with a high-risk score (Fig. 4B). Furthermore, the CNV distribution in the two distinct groups was also examined (Fig. 4C). The amplification of *CDK4, MDM2, and HMGA2* genes and the deletion of the *NF1* gene showed significantly higher prevalence in the high-risk score group than in the low-risk score group (Fig. 4D). Overall, the high-risk score patients were prone to have more gene alterations (Fig. 4D).

**Figure 5.**
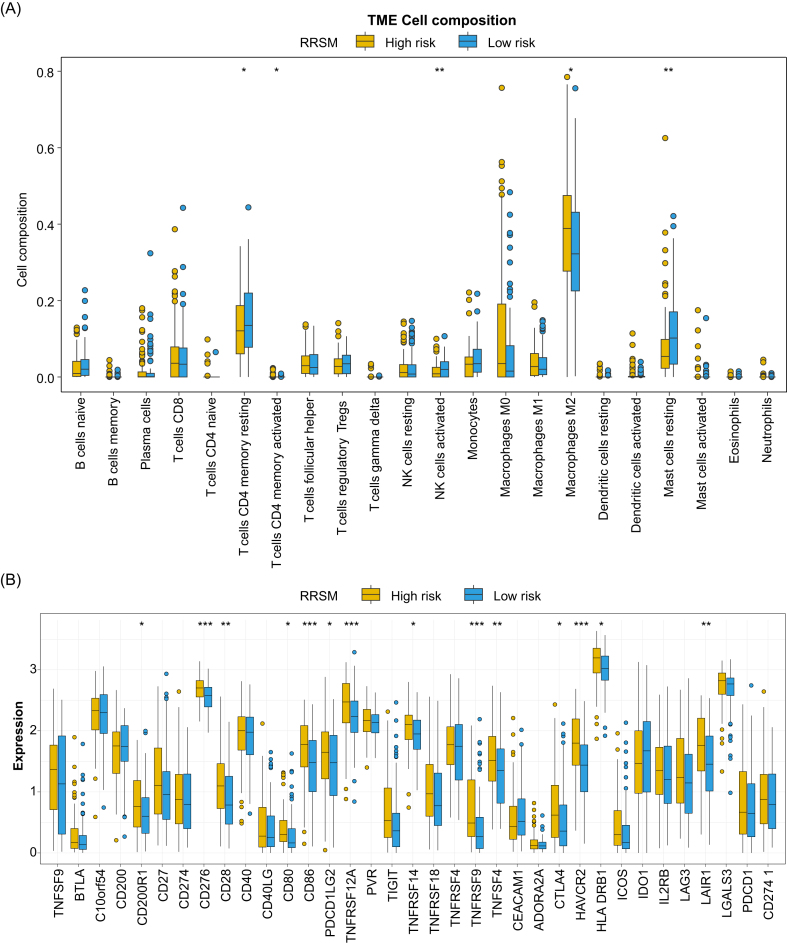
Infiltration of 22 immune cell types compared between high- and low-risk groups of patients in the training set (**A**). Expression of immune checkpoint-related genes compared between high- and low-risk groups of patients in the training set (**B**).

We next compared the infiltration of immune cells between high- and low-risk score groups, identifying 22 immune cell types using the CIBERSORT algorithm. The results revealed that M2 macrophages and resting mast cells were more prevalent in the high-risk group, while resting memory CD4 T cells, activated memory CD4 T cells, and activated natural killer cells were more abundant in the low-risk group (Fig. 5A). In addition, we observed a positive correlation between the expression of most immune checkpoint genes and the risk score (Fig. 5B). The overexpression of co-stimulator- or co-inhibitor-related genes in the high-risk group suggested potential mechanisms for immune evasion, driven by upregulation of immune checkpoint genes [26].

### Construction of the nomogram predicts the prognosis of STS

**Figure 6.**
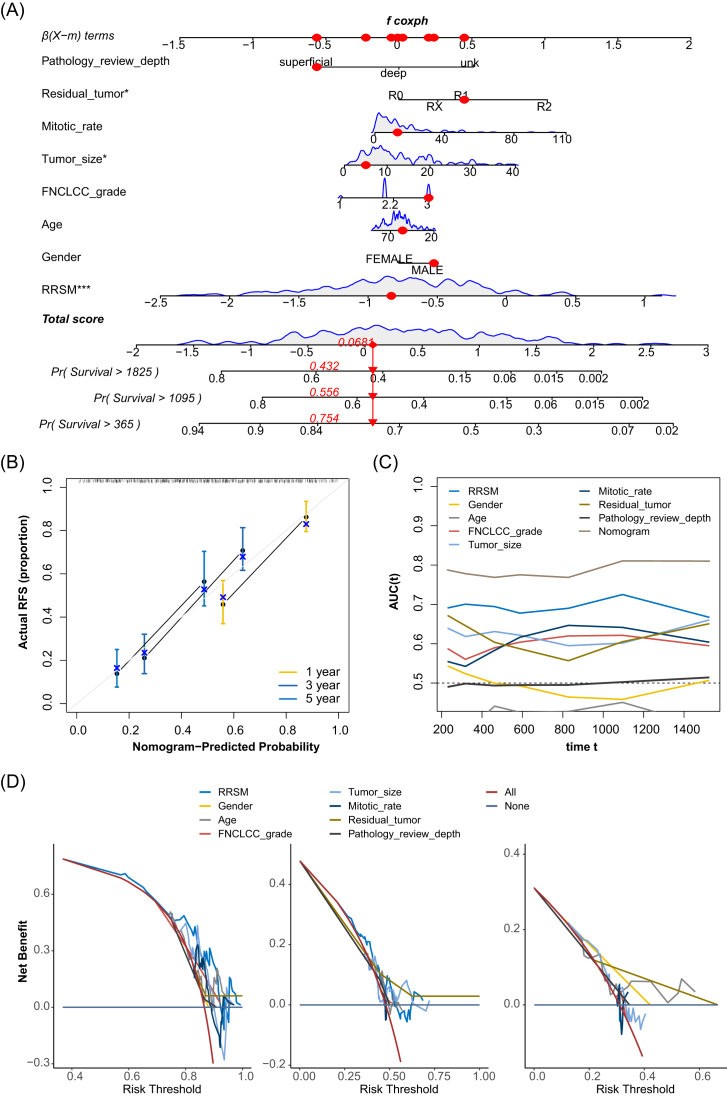
Nomogram constructed in the TCGA STS cohort. The nomogram is based on risk score, gender, age, FNCLCC grade, tumor size, mitotic rate, residual tumor, and pathology review depth (**A**). Calibration curves for the internal verification in 1-, 3-, and 5-year RFS of the nomogram (**B**). ROC curves for the predictive accuracy in 1-, 3-, and 5-year RFS of the nomogram (**C**). Nomogram net benefit analysis at 1-, 3-, and 5-year RFS intervals (**D**).

To assess the clinical utility of RRSM, we constructed a prognostic nomogram with important clinical factors (gender, age, FNCLCC (Fédération Nationale des Centres de Lutte Contre le Cancer) grade, tumor size, mitotic rate, margin of surgery resection, and RRSM) to predict patients’ RFS (Fig. 6A). By summing the points assigned to each variable, the total score generated by the nomogram estimates the likelihood of disease relapse at 1-, 3-, and 5-year intervals. Moreover, the calibration curves revealed a strong concordance between the nomogram’s predicted relapse probabilities and the actual observed outcomes at 1-, 3-, and 5-years (Fig. 6B). The AUCs demonstrated that the nomogram outperformed individual prediction based on the RRSM, gender, FNCLCC grade, and margin of surgery resection alone in forecasting RFS (Fig. 6C). Additionally, the net benefit analysis for 1-, 3-, and 5-year RFS showed that the nomogram provided superior predictive accuracy to other independent factors (Fig. 6D). Collectively, these findings underscore the nomogram’s robust predictive performance for prognosis and its enhanced applicability for STS patients.

### Drug sensitivity analysis based on the RRSM

**Figure 7.**
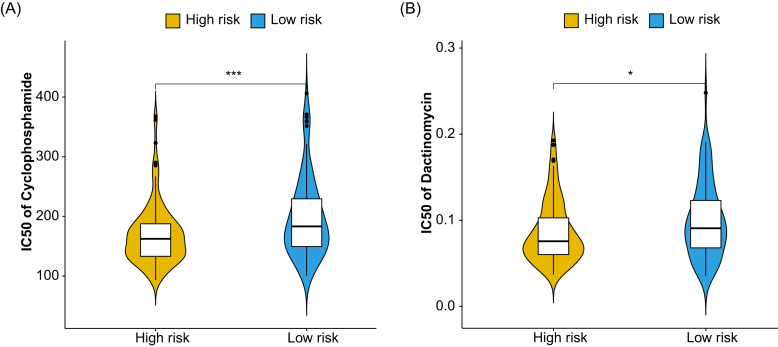
Drug sensitivity. Violin plot of the IC50 value of the two drugs, cyclophosphamide (**A**) and dactinomycin (**B**) in the TCGA STS cohort. The horizontal axes are the high- and low-risk groups, and the vertical axes represent the IC50 values.***P < 0.001；*P < 0.05.

Adjuvant chemotherapy has been reported to improve local and distant RFS in extremity STS [27]. Recognizing the critical role of adjuvant chemotherapy in STS management, we investigated the association between RRSM and sensitivity to commonly used anticancer drugs for STS. We compared the IC50 values, an indicator of drug sensitivity, between the high- and low-risk score groups. Both cyclophosphamide and dactinomycin exhibited increased sensitivity in patients with high-risk scores (Fig. 7A and B). This result indicated that RRSM could serve as a predictive biomarker in guiding STS treatment.

### *In vivo* validation of the genes in the RRSM

**Figure 8.**
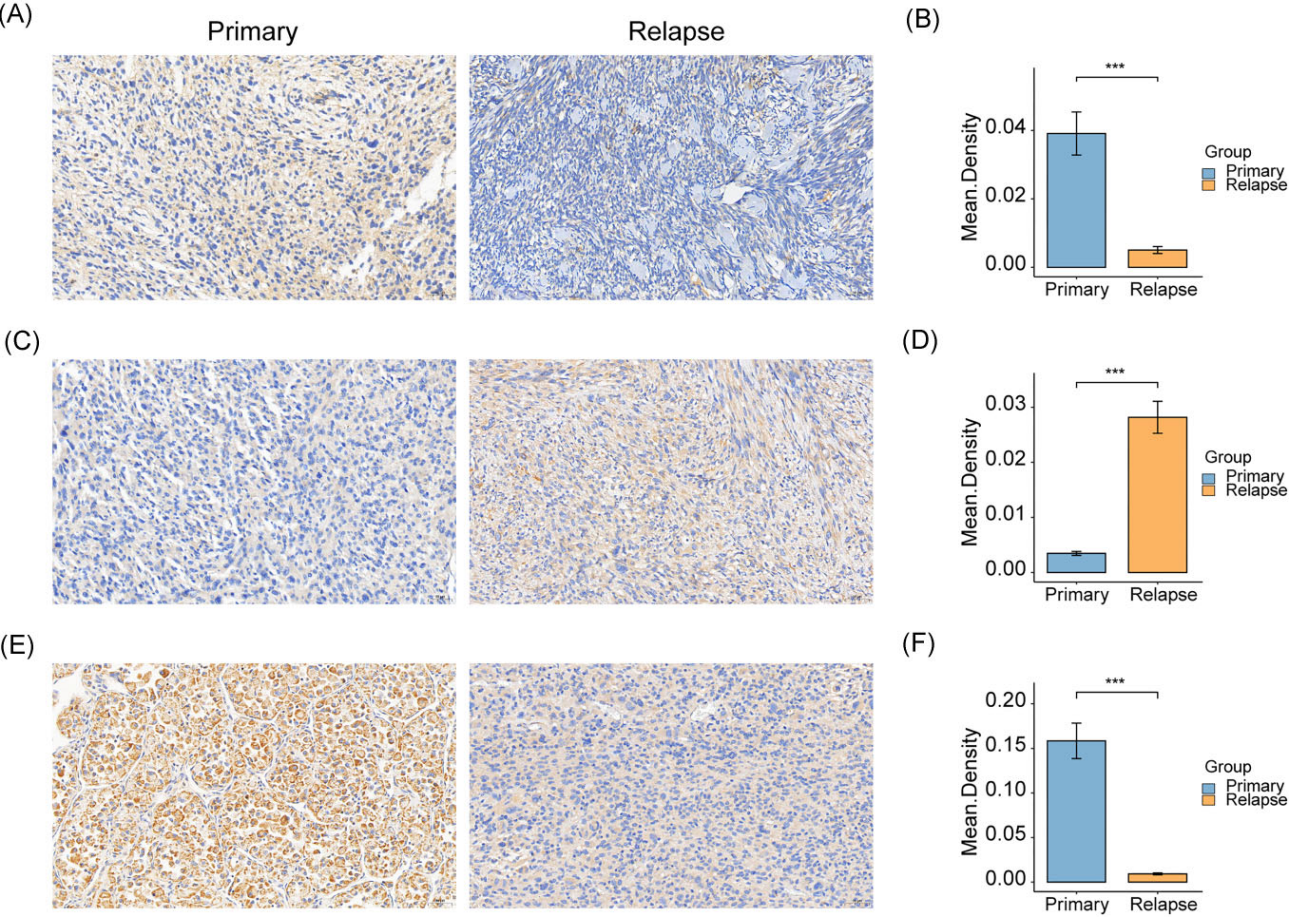
Immunohistochemical analysis of genes in the RRSM in primary and relapsed STS. Immunohistochemical staining for FZD7 (**A**), ITPKA (**C**), and PRKAG1 (**E**). Comparative analysis of FZD7 (**B**), ITPKA (**D**), and PRKAG1 (**F**) expression. Error bars represent the standard error of the mean (SEM). The statistical significance was determined using a Wilcoxon test, and the results are presented as the mean density of staining. The asterisks indicate the level of statistical significance (***P < 0.001).

To further validate the expression status of the genes involved in the construction of the RRSM, we performed immunohistochemistry in the tissue sections of relapsed and primary STS patients. The results showed that the expression levels of FZD7 and PRKAG1 were reduced in relapsed tumors compared with primary ones, while ITPKA exhibited elevated levels in relapsed tumors (Fig. 8). However, no significant difference in COL6A3 expression was observed between the two groups.

## Discussion

Relapsed STS patients face poor outcomes and have limited treatment options [7]. Overall, the TME in relapsed STS was markedly different from that in primary STS, contributing to the poor prognosis. This study leverages multiplatform profiling to compare relapsed versus primary STS, identifying several events derived from genomic, transcriptomic, and epigenetic features that may explain the recurrence mechanism. An RRSM for STS prognosis was constructed using genes in relapse-associated sub-pathways. The FZD7, PRKAG1, and ITPKA genes, as potential therapeutic targets, may aid in STS patient prognosis classification and clinical management.

In this study, we compared the multi-omic profiles between relapsed and primary extremity and trunk STS patients. The genomic profile analysis revealed that extremity and trunk STS had fewer mutations and more CNVs, consistent with previous studies. Alterations in the RAD51C gene and mutations in the Wnt pathway were more frequently detected in relapsed STS. Lee *et al*. demonstrated that metastatic breast cancer patients with RAD51C amplification had significantly shorter progression-free survival (PFS) (≤ 6 months) than long PFS (> 6 months), correlating with poor prognosis [28]. Activation of the WNT signaling pathway has been reported in relapsed small-cell lung cancer [29]. The finding suggested that RAD51C gene alterations (somatic mutation and amplification) and Wnt signaling pathway activation may be involved in the recurrence of extremity and trunk STS.

Transcriptome profile analysis suggested that the relapsed STS was prone to create a pro-tumor microenvironment characterized by cell junction assembly, extracellular structure organization, collagen-containing extracellular matrix, growth factor binding, epithelial–mesenchymal transition, and notch signaling. In contrast, primary STS exhibited an immune-activated TME with high levels of leukocyte migration/chemotaxis, (positive) regulation of leukocyte migration/chemotaxis, cytokine binding, chemokine binding, inflammatory response, and fatty acid metabolism. Yang *et al*. reported that stage IA lung adenocarcinoma (LUAD) patients without relapse had significantly higher expression of chemokine, cytolytic activity, and tumor-associated antigen gene signature than relapsed LUAD patients [30]. A study from the AURORA US Network suggested that primary tumors had markedly higher expression of adaptive immune features than metastases in metastatic breast cancer, with immune-related signatures predicting better outcomes [31]. Numerous studies confirmed that fatty acid metabolism could promote cancer cell angiogenesis, migration, and invasion [32]. Moreover, fatty acid metabolism regulates the function and differentiation of immune cells in the TME, thus targeting fatty acid metabolism could synergize with immunotherapy and promote anti-tumor immunity [33]. The transcriptome profiles confirmed the significant differences in TME between primary and relapsed STS.

Utilizing the TCGA data, we constructed the RRSM for STS prognosis using genes in the relapse-associated sub-pathway. The model could identify the prognosis for both training and validation cohorts. The function and prognostic implications of the four genes in the RRSM classifier have been discussed in previous studies but not in soft tissue cancer. The expression of COL6A3 was up-regulated in cancer, and its silencing can inhibit cancer cell proliferation, angiopoiesis, migration, invasion, and apoptosis [34, 35]. High COL6A3 expression has been associated with poor prognosis across various tumors [36–38]. In the present study, although we did not find different expression of COL6A3 between primary and relapsed STS, its continuous expression level when selected by LASSO carries independent prognostic value. FZD7 has been implicated in tumor aggressiveness through the Wnt/PCP pathway in ovarian cancer [39], and its upregulation during melanoma progression suggests a role in inducing tumorigenesis and tumor growth by WNT11-FZD7-DAAM1 signaling [40]. Inositol-1,4,5-triphosphate-3-kinase-A (ITPKA) plays an important role in regulating calcium signaling and actin dynamics, and its dysregulation has been reported in multiple cancers [41, 42]. ITPKA overexpression in LUAD contributed to the malignant phenotypes by promoting EMT progression and interacting with Drebrin1 [43]. PRKAG1 encodes the AMPKγ1 subunit, a core component of the AMPK signaling pathway, and its phosphorylation regulates AMPK localization and activity [44]. Further investigation into the potential mechanisms of these RRSM-based genes in tumorigenesis and progression is warranted. Although our findings suggest these genes may contribute to recurrence risk in STS, the precise molecular mechanisms remain to be elucidated. Future *in vitro* and *in vivo* functional studies will be required to clarify how these genes regulate tumor invasion, metastasis, and immune evasion.

The RRSM can effectively stratify STS patients into high- and low-risk groups, with the high-risk group exhibiting a significantly shorter RFS in both the training and validation cohorts. Comprehensive analyses, including gene alteration analysis, immune cell infiltration analysis, and immune checkpoint gene analysis were performed to elucidate the possible molecular mechanism of the effect of RRSM on STS prognosis. High-risk score patients had higher genetic alteration frequency in oncogenes and tumor suppressor genes. SMARCAL1, a member of the SNF2 family, is crucial for maintaining genome stability and regulating gene expression [45–48]. SMARCAL1-deficient breast cancer cell lines could enhance the response to immunotherapy [49]. B4GALNT1 promotes metastasis and EMT by activating the JNK/c-Jun/Slug pathway in LUAD [50]. The dysfunction of type I interferon signaling can faciliate immune escape and tumor metastasis [51–53]. Alterations and mutations of OBSCN were detected in many solid tumors [54–56], with higher OBSCN expression levels predicting poor survival outcomes in papillary renal cell carcinoma (pRCC) [57]. NOS1 promotes lung metastasis by inducing dysfunctional interferon signaling in melanoma [58]. Our study suggested that SMARCAL1 mutation was significantly more prevalent in the low-risk group than in the high-risk group. The amplification of several oncogenes, mainly CDK4, MDM2, and HMGA2, was involved in liposarcoma pathogenesis [59]. As expected, our study found that high-risk score STS patients had a significantly higher frequency of CDK4/MDM2/HMGA2 amplification than those with low-risk score. NF1, a tumor suppressor gene that negatively regulates Ras signaling, was identified as one of the recurrent deletion genes in dedifferentiated liposarcoma [11]. Deletion of NF1 was significantly more common in the high-risk score group than in the low-risk score group. In addition, a comprehensive characterization of cell-infiltrating in the TME revealed that the low-risk score group had significantly higher immune-activated cell subtype infiltration, including activated CD4 memory T cells and activated natural killer cells, while the high-risk score patients exhibited higher infiltration of pro-tumorigenesis M2 macrophages. Moreover, the overexpression of both co-stimulatory and co-inhibitory genes was detected in the high-risk score group, implying possible immune evasion mechanisms through the overexpression of immune checkpoint genes [26]. These findings suggest that genetic instability and immune evasion jointly underlie the poor prognosis of high-risk STS patients. Moreover, high-risk patients demonstrated increased sensitivity to cyclophosphamide and dactinomycin, pointing to the potential therapeutic relevance of the RRSM. The above-mentioned molecular differences between the high-risk score and low-risk score groups could partially explain the mechanism of RRSM affecting the prognosis.

Notably, high-risk patients showed increased sensitivity to cyclophosphamide and dactinomycin. This may reflect genomic vulnerabilities in high-risk tumors, including CDK4/MDM2/HMGA2 amplifications and NF1 deletions, which impair DNA damage response and heighten transcriptional stress [60–62]. The immunosuppressive TME of high-risk STS may further potentiate chemotherapy efficacy by reducing immune-mediated resistance [63]. Clinically, RRSM could identify patients most likely to benefit from intensified or combinatorial regimens (e.g. cyclophosphamide with immune checkpoint inhibitors). Prospective studies are warranted to validate these predictive associations.

The limitations of this study should also be recognized. First, the modest size of our in-house cohort (*n* = 35) may introduce statistical bias and limit generalizability. To minimize this, we used these cases only for feature discovery, while the RRSM was constructed in the larger TCGA cohort (*n* = 206) and validated across three external GEO datasets (>700 cases). Nevertheless, future work should include larger, multi-center cohorts with broader subtype representation to enhance robustness and clinical applicability. Second, due to the limited sample size of individual subtypes in both our in-house and TCGA cohorts, we adopted a pan-STS approach. While this strategy maximizes statistical power, it may overlook subtype-specific recurrence mechanisms. We acknowledge that extremity and trunk STS encompass diverse histological subtypes (e.g. liposarcoma, leiomyosarcoma, synovial sarcoma, undifferentiated pleomorphic sarcoma), each with distinct molecular alterations and clinical behaviors. Future studies with larger and multi-center cohorts are needed to perform subtype-focused analyses, which may further refine the prognostic precision of the RRSM. In addition, because the current model relied solely on transcriptomic data, it may not fully capture the molecular complexity underlying STS recurrence. Incorporating mutation and methylation data in future multi-omics analyses may further enhance the biological interpretability and predictive performance of the model. Third, we noted that GSE71119 yields an AUC of 0.42, which diverges from the 0.63–0.64 observed in the two larger validation sets. The small cohort size and imbalance between risk groups likely result in this unstable performance of RRSM. Only 23 of the 47 events were classified as high risk, resulting in limited discriminatory leverage and heightened sensitivity to sampling fluctuations. By contrast, the two larger validation sets (GSE98765 and GSE54321) maintained balanced risk strata. Their AUCs of 0.63 and 0.64 therefore offer a more reliable estimate of the model’s generalizability. Taken together, these observations suggest that the apparent drop in GSE71119 reflects limited statistical power rather than a fundamental deficiency of the model. The concordant performance in the larger validation cohorts indicates that, when adequate sample sizes are available, the RRSM retains clinically useful discrimination. Future applications should therefore prioritize sufficiently powered datasets, while ongoing efforts to enhance biological anchoring remain warranted to further improve robustness across heterogeneous populations.

In this study, we investigated the multi-omic features between relapsed and primary STS that underwent operation resection. Our analysis revealed that primary STS exhibits an immune-hot microenvironment. Subsequently, we developed an RRSM that could predict the prognosis in the TCGA and GEO STS cohorts. In addition, we validated expression of the four genes in tumor tissue in primary and relapsed STS patients, providing potential treatment targets for STS patients. Due to the limited sample size in our study, future investigations must enroll a larger number of STS patients to enhance the robustness of our findings and to further explore the clinical implications of our risk score model.

## Supplementary Material

pbaf027_Supplemental_Files
