# Supplementary material for: The recurrence or metastasis related gene predicts the prognosis of extremity and trunk soft tissue sarcoma
Source: Precis Clin Med. 2025 Oct 28;8(4):pbaf027. doi: 10.1093/pcmedi/pbaf027 (PMC12793454; doi:10.1093/pcmedi/pbaf027)
Supplement: pbaf027_Supplemental_Files [file pbaf027_Supplemental_Files.zip › Supplementary figures.pdf]

Supplementary Figures

Supplementary Figure 1. The violin plot of TMB values was calculated between primary and relapsed STS patients (A). The boxplot of gene fusion detected in primary and relapsed STS patients (B). Oncoplot of 91 diagnostic outpatient STS cases, split by fusion status (blue = with fusion, green = without). The matrix shows mutations/CNVs; the side bar lists the 20 most altered genes (C). Patterns of mutational process of 36 extremity and trunk STS patients. X-axes show the mutation types, and the percentage of mutations in the signature attributed to each mutation are displayed on the Y axes (D). The column plot of signature exposures showing difference between primary and relapsed STS patients (E).

Supplementary Figure 2. The GO enrichment and hallmark enrichment analysis of upregulated genes (A) and down-regulated genes (B) in relapsed STS. Dot plots of biological process (BP), cellular components (CC), and molecular function (MF) in GO enrichment analysis. The horizontal coordinate is generated, representing the ratio of a term's genes to the total genes. The vertical coordinate is the name of the enriched terms. The color represents the p-value, the redder the color the larger the p-value. The cancer hallmark pathway analysis showed activated (C) and suppressed (D) pathways in the relapsed group. The difference of 22 immune cell infiltration levels between relapsed and primary STS patients (E).

Supplementary Figure 3. The GO enrichment and hallmark enrichment analysis of different expressed genes (DEGs). Dot plots of cellular components, molecular function, and biological process in GO enrichment analysis. The horizontal coordinate is generated, representing the ratio of a term's genes to the total genes. The vertical coordinate is the name of the enriched terms. The color represents the p-value, the redder the color the larger the p-value.

Supplementary Figure 4. Kaplan-Meier survival analysis for the RFS of COL6A3 (A), FZD7 ITPKA (B), FZD7 (C), and PRKAG1 (D).

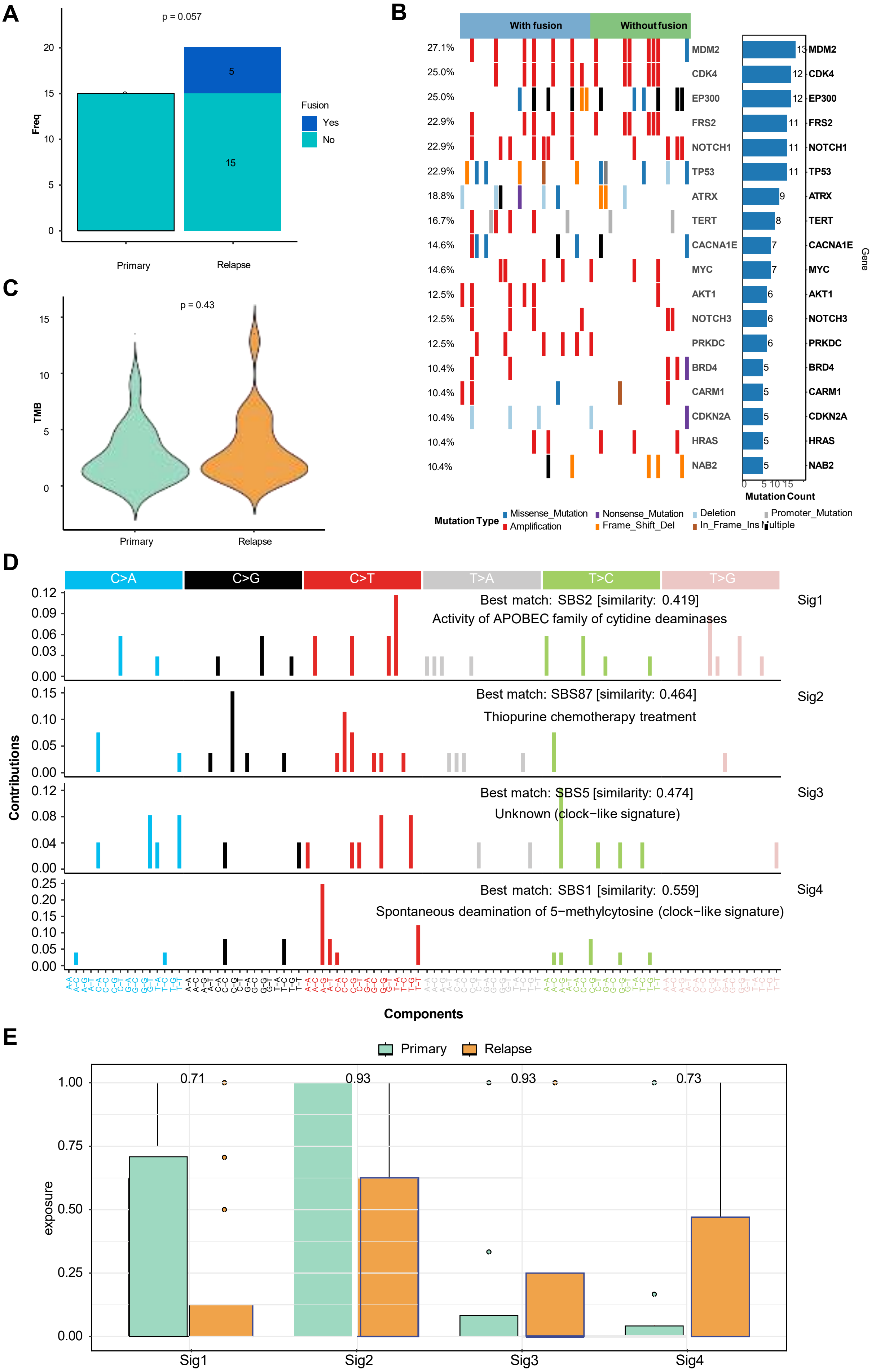

A
p = 0.057
Freq
Primary
Relapse
Fusion
Yes
No
5
15
B
With fusion
Without fusion
MDM2
CDK4
EP300
FRS2
NOTCH1
TP53
ATRX
TERT
CACNA1E
MYC
AKT1
NOTCH3
PRKDC
BRD4
CARM1
CDKN2A
HRAS
NAB2
Mutation Count
Gene
Mutation Type
Missense_Mutation
Nonsense_Mutation
Deletion
Promoter_Mutation
Amplification
Frame_Shift_Del
In_Frame_Ins
Multiple
C
p = 0.43
TMB
Primary
Relapse
D
C>A
C>G
C>T
T>A
T>C
T>G
Best match: SBS2 [similarity: 0.419]
Activity of APOBEC family of cytidine deaminases
Sig1
Best match: SBS87 [similarity: 0.464]
Thiopurine chemotherapy treatment
Sig2
Best match: SBS5 [similarity: 0.474]
Unknown (clock−like signature)
Sig3
Best match: SBS1 [similarity: 0.559]
Spontaneous deamination of 5−methylcytosine (clock−like signature)
Sig4
Contributions
Components
E
Primary
Relapse
exposure
0.71
0.93
0.93
0.73
Sig1
Sig2
Sig3
Sig4

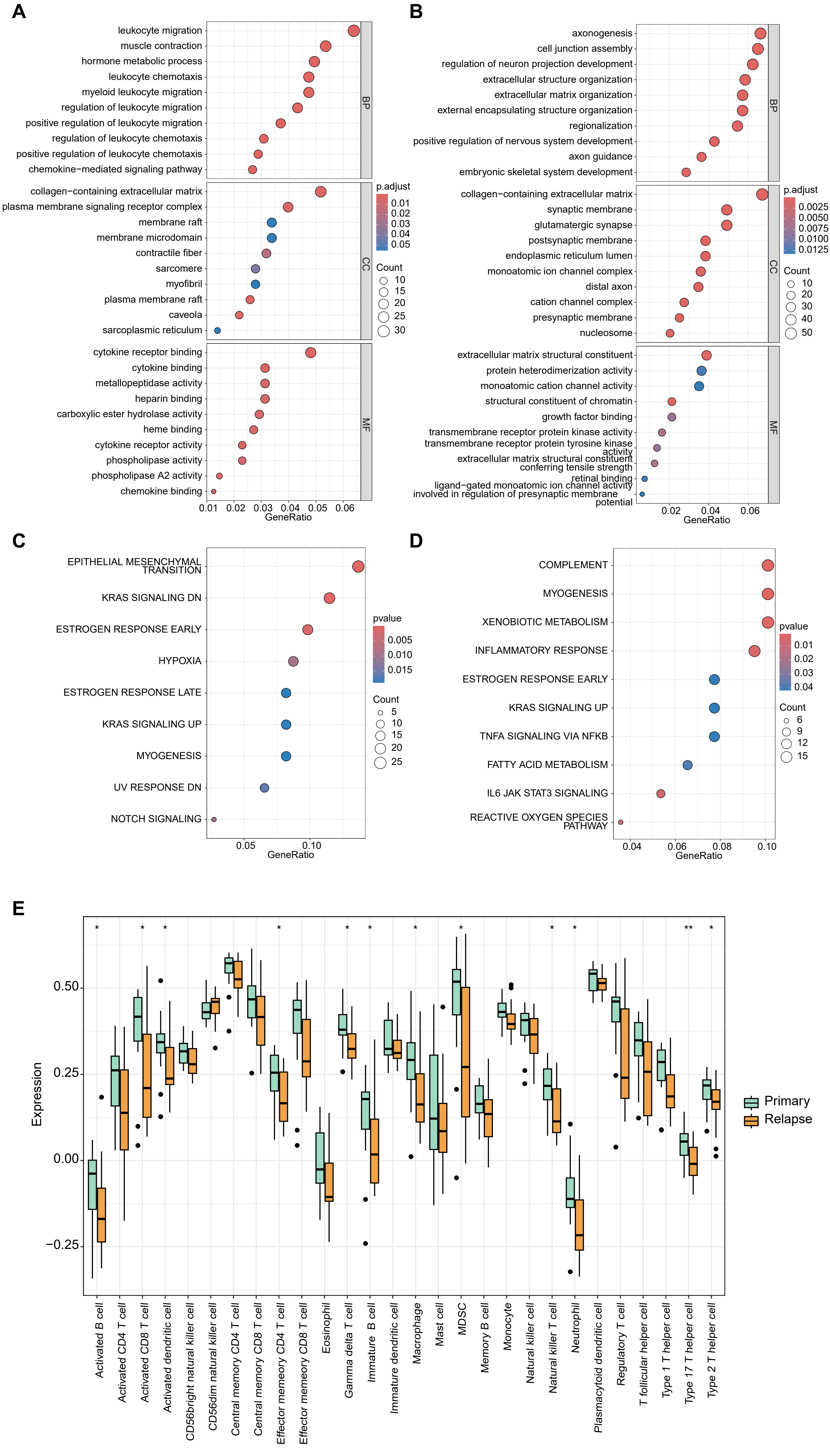
A
leukocyte migration
muscle contraction
hormone metabolic process
leukocyte chemotaxis
myeloid leukocyte migration
regulation of leukocyte migration
positive regulation of leukocyte migration
regulation of leukocyte chemotaxis
positive regulation of leukocyte chemotaxis
chemokine−mediated signaling pathway
collagen−containing extracellular matrix
plasma membrane signaling receptor complex
membrane raft
membrane microdomain
contractile fiber
sarcomere
myofibril
plasma membrane raft
caveola
sarcoplasmic reticulum
cytokine receptor binding
cytokine binding
metallopeptidase activity
heparin binding
carboxylic ester hydrolase activity
heme binding
cytokine receptor activity
phospholipase activity
phospholipase A2 activity
chemokine binding
BP
CC
MF
GeneRatio
p.adjust
Count
B
axonogenesis
cell junction assembly
regulation of neuron projection development
extracellular structure organization
extracellular matrix organization
external encapsulating structure organization
regionalization
positive regulation of nervous system development
axon guidance
embryonic skeletal system development
collagen−containing extracellular matrix
synaptic membrane
glutamatergic synapse
postsynaptic membrane
endoplasmic reticulum lumen
monoatomic ion channel complex
distal axon
cation channel complex
presynaptic membrane
nucleosome
extracellular matrix structural constituent
protein heterodimerization activity
monoatomic cation channel activity
structural constituent of chromatin
growth factor binding
transmembrane receptor protein kinase activity
transmembrane receptor protein tyrosine kinase activity
extracellular matrix structural constituent conferring tensile strength
retinal binding
ligand−gated monoatomic ion channel activity involved in regulation of presynaptic membrane potential
GeneRatio
p.adjust
Count
C
EPITHELIAL MESENCHYMAL TRANSITION
KRAS SIGNALING DN
ESTROGEN RESPONSE EARLY
HYPOXIA
ESTROGEN RESPONSE LATE
KRAS SIGNALING UP
MYOGENESIS
UV RESPONSE DN
NOTCH SIGNALING
GeneRatio
pvalue
Count
D
COMPLEMENT
MYOGENESIS
XENOBIOTIC METABOLISM
INFLAMMATORY RESPONSE
ESTROGEN RESPONSE EARLY
KRAS SIGNALING UP
TNFA SIGNALING VIA NFKB
FATTY ACID METABOLISM
IL6 JAK STAT3 SIGNALING
REACTIVE OXYGEN SPECIES PATHWAY
GeneRatio
pvalue
Count
E
Expression
Primary
Relapse
Activated B cell
Activated CD4 T cell
Activated CD8 T cell
Activated dendritic cell
CD56bright natural killer cell
CD56dim natural killer cell
Central memory CD4 T cell
Central memory CD8 T cell
Effector memeory CD4 T cell
Effector memeory CD8 T cell
Eosinophil
Gamma delta T cell
Immature B cell
Immature dendritic cell
Macrophage
Mast cell
MDSC
Memory B cell
Monocyte
Natural killer cell
Natural killer T cell
Neutrophil
Plasmacytoid dendritic cell
Regulatory T cell
T follicular helper cell
Type 1 T helper cell
Type 17 T helper cell
Type 2 T helper cell

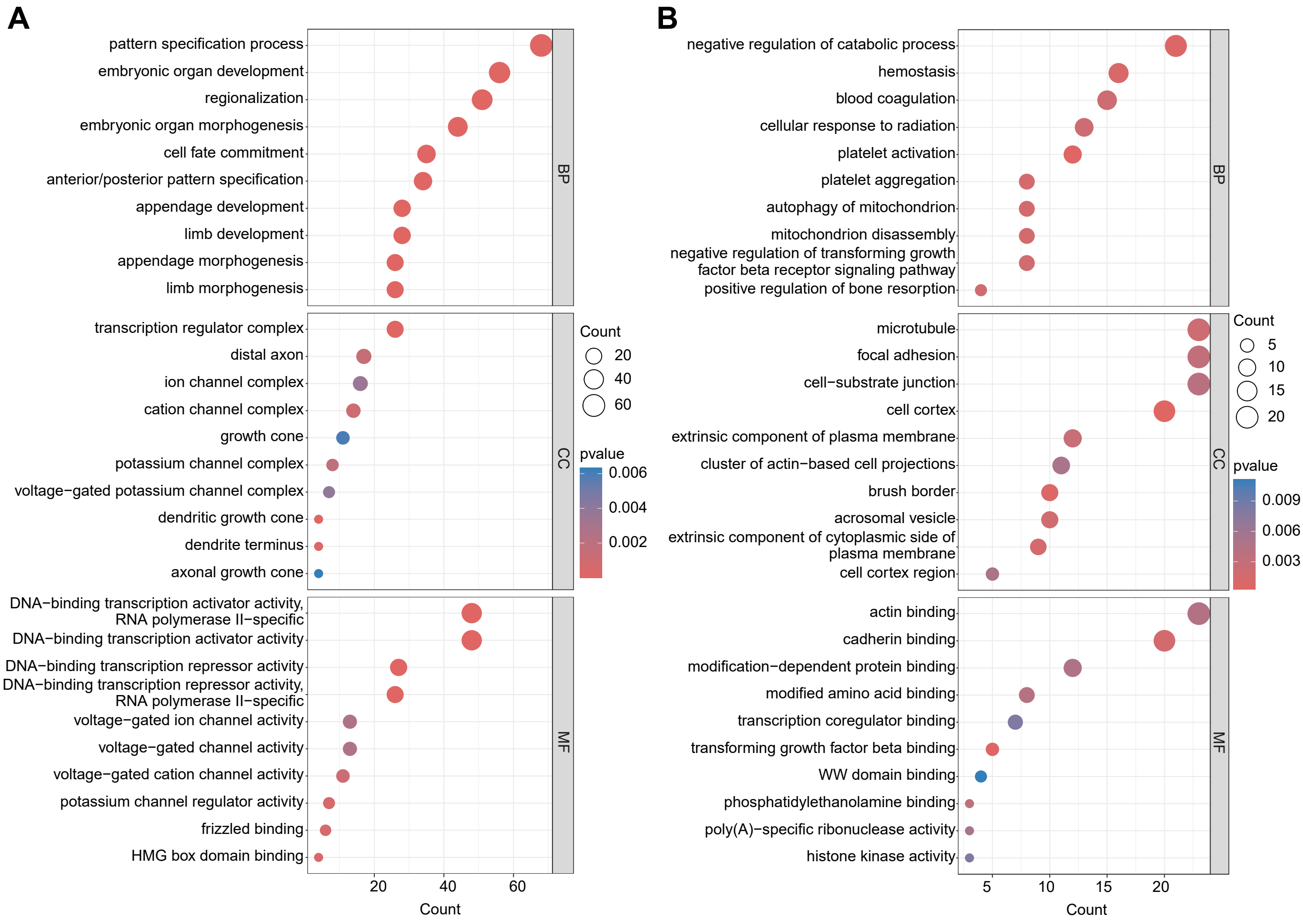
A
pattern specification process
embryonic organ development
regionalization
embryonic organ morphogenesis
cell fate commitment
anterior/posterior pattern specification
appendage development
limb development
appendage morphogenesis
limb morphogenesis
transcription regulator complex
distal axon
ion channel complex
cation channel complex
growth cone
potassium channel complex
voltage−gated potassium channel complex
dendritic growth cone
dendrite terminus
axonal growth cone
DNA−binding transcription activator activity, RNA polymerase II−specific
DNA−binding transcription activator activity
DNA−binding transcription repressor activity
DNA−binding transcription repressor activity, RNA polymerase II−specific
voltage−gated ion channel activity
voltage−gated channel activity
voltage−gated cation channel activity
potassium channel regulator activity
frizzled binding
HMG box domain binding
BP
CC
MF
20
40
60
Count
Count
20
40
60
pvalue
0.006
0.004
0.002
B
negative regulation of catabolic process
hemostasis
blood coagulation
cellular response to radiation
platelet activation
platelet aggregation
autophagy of mitochondrion
mitochondrion disassembly
negative regulation of transforming growth factor beta receptor signaling pathway
positive regulation of bone resorption
microtubule
focal adhesion
cell−substrate junction
cell cortex
extrinsic component of plasma membrane
cluster of actin−based cell projections
brush border
acrosomal vesicle
extrinsic component of cytoplasmic side of plasma membrane
cell cortex region
actin binding
cadherin binding
modification−dependent protein binding
modified amino acid binding
transcription coregulator binding
transforming growth factor beta binding
WW domain binding
phosphatidylethanolamine binding
poly(A)−specific ribonuclease activity
histone kinase activity
BP
CC
MF
5
10
15
20
Count
Count
5
10
15
20
pvalue
0.009
0.006
0.003

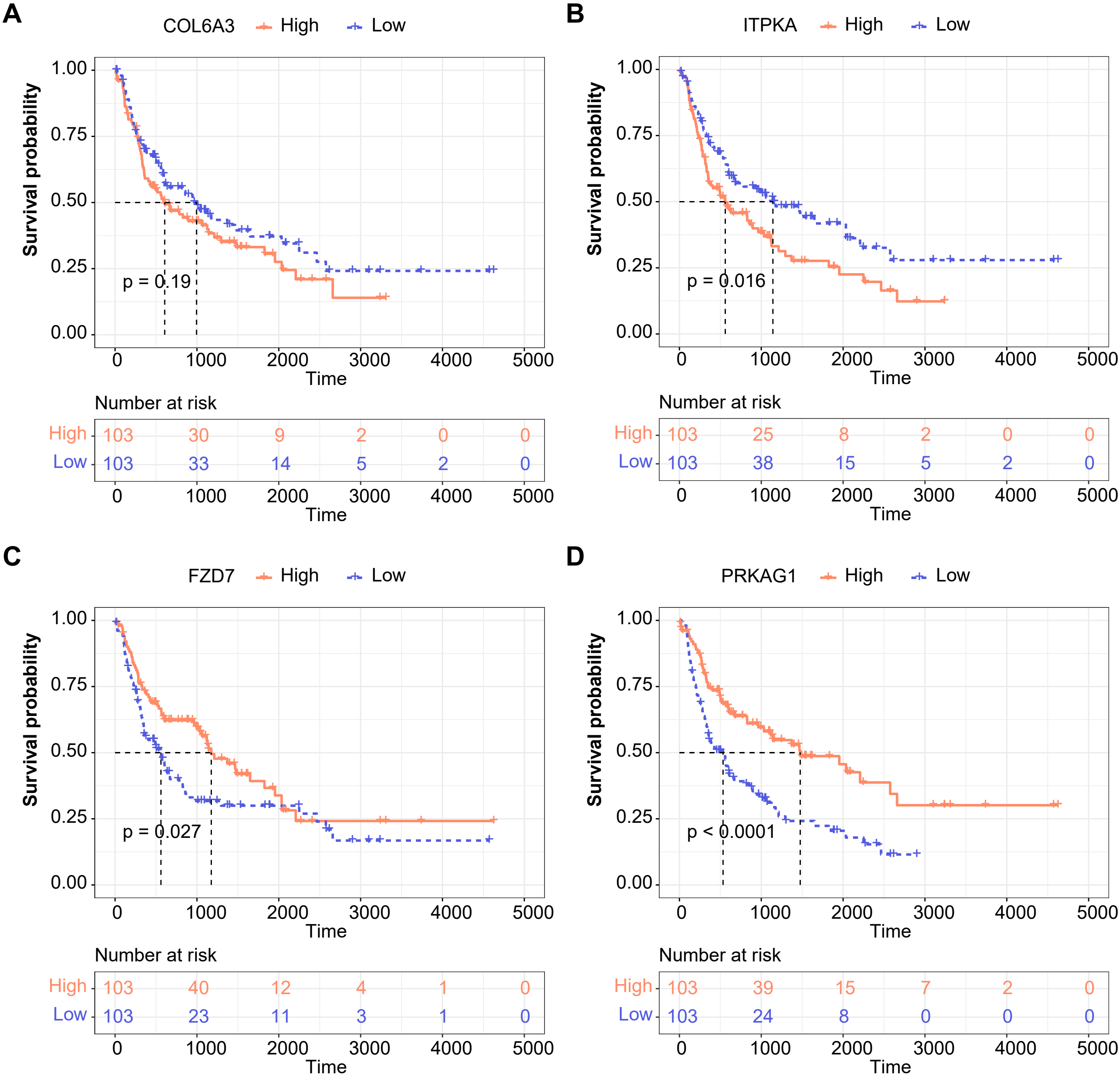
A
COL6A3 High Low
Survival probability
1.00
0.75
0.50
0.25
0.00
p = 0.19
0 1000 2000 3000 4000 5000
Time
Number at risk
High 103 30 9 2 0 0
Low 103 33 14 5 2 0
0 1000 2000 3000 4000 5000
Time
B
ITPKA High Low
Survival probability
1.00
0.75
0.50
0.25
0.00
p = 0.016
0 1000 2000 3000 4000 5000
Time
Number at risk
High 103 25 8 2 0 0
Low 103 38 15 5 2 0
0 1000 2000 3000 4000 5000
Time
C
FZD7 High Low
Survival probability
1.00
0.75
0.50
0.25
0.00
p = 0.027
0 1000 2000 3000 4000 5000
Time
Number at risk
High 103 40 12 4 1 0
Low 103 23 11 3 1 0
0 1000 2000 3000 4000 5000
Time
D
PRKAG1 High Low
Survival probability
1.00
0.75
0.50
0.25
0.00
p < 0.0001
0 1000 2000 3000 4000 5000
Time
Number at risk
High 103 39 15 7 2 0
Low 103 24 8 0 0 0
0 1000 2000 3000 4000 5000
Time
